# Association between *Helicobacter pylori* infection and dental pulp reservoirs in Japanese adults

**DOI:** 10.1186/s12903-019-0967-2

**Published:** 2019-12-02

**Authors:** Komei Iwai, Isao Watanabe, Toshiro Yamamoto, Nagato Kuriyama, Daisuke Matsui, Ryota Nomura, Yuko Ogaya, Fumishige Oseko, Keiji Adachi, Shigeta Takizawa, Etsuko Ozaki, Teruhide Koyama, Kazuhiko Nakano, Narisato Kanamura, Ritei Uehara, Yoshiyuki Watanabe

**Affiliations:** 1 Department of Dental Medicine, Kyoto Prefectural University of Medicine, Graduate School of Medical Science, 465 Kajiimachi, Kamigyoku, Kyoto, 602-8856 Japan; 2 Department of Epidemiology for Community Health and Medicine, Kyoto Prefectural University of Medicine, Graduate School of Medical Science, Kyoto, Japan; 3 Department of Pediatric Dentistry, Division of Oral Infection and Disease Control, Osaka University Graduate School of Dentistry, Osaka, Japan

**Keywords:** *Helicobacter pylori* infection, *H. pylori*, Ulcer, Gastric cancer, Dental pulp, Dental caries, Dentistry

## Abstract

**Background:**

*Helicobacter pylori* (*H. pylori*) colonize the stomach and are considered an etiological agent of gastric cancer. The oral cavity is a transmission route to the stomach, but the exact site of colonization has not yet been explicated. Our study investigated the association between *H. pylori* infection and presence in oral samples.

**Methods:**

Dental pulp, supragingival plaque, and saliva from 192 patients visiting the Dentistry’s outpatient clinic were collected for testing. The *H. pylori ureA* gene was identified via Nested PCR. Urine anti-*H. pylori* antibody test was utilized to detect infection.

**Results:**

Twenty-five subjects were found to be antibody-positive. PCR analysis of dental pulp revealed that 23 subjects possessed the *ureA* gene. Twenty-one subjects were positive for both antibodies and genes in dental pulp. PCR testing revealed that 2 subjects were positive in dental plaque but negative for saliva. The subjects positive for *H. pylori* in dental pulp expressed clinical signs of severe dental caries.

**Conclusions:**

*H. pylori* infected subjects expressed *H. pylori* in samples from the oral cavity. The main reservoir for infection within the oral cavity was determined to be dental pulp. Moreover, *H. pylori* are likely transmitted from dental caries to the root canal.

## Background

*Helicobacter pylori* existed for a very long time as the etiological agent of ulcers but was not recognized as such, until 1983, when Warren and Marshall went against the scientific ideology of the time and announced that ulcers were caused by bacteria, not stress [1]. *H. pylori* is a gram-negative, microaerophilic, motile rod, with a distinctive spiral shape, and long flagella that assist with both motility and attachment within the stomach mucosal layer. This bacterium causes chronic gastritis and duodenal ulcers, and produces the enzyme urease, which neutralizes gastric acid locally, enabling colonization of the stomach. In addition, pathogenic factors such as *cagA and vacA* produced by *H. pylori* have been found to produce a pronounced inflammatory reaction within the stomach. Additionally, *H. pylori* are carcinogenic bacteria and infection may result in gastric cancer [2]. Moreover, more than half of the world population is infected with *H. pylori* [3]. Further, incidence of *H. pylori* infection was much higher in unhygienic or socioeconomically disadvantaged regions. Correspondingly, the proportion of *H. pylori* infections in Asia, Africa, and South America was significantly higher than that of North America, Western Europe, or Australia [4]. In Japan, The proportion of *H. pylori* infection among the Japanese was approximately 28% from 2008 to 2012 and more than 68% in individuals aged 70 years and over, 46% in individuals in their 60s, and 37% in individuals in their 50s [5]. Hence, the proportion of infection was found to be lower in younger generations.

The transmission route is assumed to be either oral-oral infection or fecal-oral infection [6]. To date, *H. pylori* has been detected in samples of dental plaque, saliva, and dental pulp [7–12]. Some studies have reported an association between *H. pylori* infection and its presence in the oral cavity. Okuda et al. reported that 12 of 54 *H. pylori*-infected subjects (22%) expressed *H. pylori* in their dental plaque [13]. Similarly, Bagot et al. reported that 12 of 56 *H. pylori*-infected subjects (21%) possessed the bacterium in their saliva [14]. Thus, the presence of *H. pylori* in the oral cavity appears to be indicative of *H. pylori* infection.

Molecular biological techniques such as PCR have been used extensively for detection of *H. pylori* genes in samples obtained from oral cavity [15–19]. In previous research, partial *H. pylori*-specific genes (*cagA*, *vacA*, *ureA*, and *ureC*) and the 16S rRNA gene were amplified. However, the proportion of *H. pylori* in oral cavity-derived samples varied by investigational area, oral samples, and PCR primers. Further, Ogaya et al. analyzed the full DNA sequence of 48 *H. pylori* strains from GenBank (http://www.ncbi.nlm.nih.gov/genbank/) and developed a PCR method that tests for *H. pylori* by specifically targeting the *ureA* gene [12]. The sensitivity threshold for this PCR primer-based method was approximately 10^2^–10^3^ colony forming units (CFU) per reaction, when *H. pylori* genomic DNA was used as a template. Moreover, Nomura et al. optimized this technique and developed a highly sensitive nested PCR assay [20]. The detection limit of the nested PCR method was approximately 1–10 CFU per reaction, when predetermined serial dilutions of *H. pylori* DNA were added to non-bacterial infected pulp samples. The study of Nomura et al. utilized nested PCR to identify *H. pylori* colonization of the dental pulp of both deciduous and permanent teeth in children (1–19 years old). However, to date, there have been no reports utilizing this method to determine the association between *H. pylori* infection and presence in the oral cavity.

Our study optimized nested PCR to investigate the presence of *H. pylori* in the dental pulp of permanent teeth, dental plaque, and saliva of Japanese adults. We also studied the association between *H. pylori* infection and the presence of *H. pylori* in samples obtained from the oral cavity.

## Methods

### Subjects

Participants were recruited among all adult patients subjected to dental pulp treatment or tooth extraction at the outpatient clinic of the Department of Dentistry, Kyoto Prefectural University of Medicine between January 2016 and February 2017. Individuals presenting with an impacted wisdom tooth were excluded from the study. Overall, 205 patients were recruited for participation. All subjects submitted written informed consent to the attending dental doctor. Of this group, 13 subjects were excluded for the following reasons failed to complete a self-administered questionnaire (2 males), refused to submit a urine sample (4 males, 2 females), or saliva sample was difficult to collect (2 males, 3 females). Therefore, 192 subjects (87 males, 105 females; 58.6 ± 18.8 years of age) were included in the final analysis.

### Survey of characteristics, oral examinations, and dental clinical diagnosis

Past medical history was evaluated for each subject via a questionnaire which related the following information lifestyle habits, previous *H. pylori* eradication therapy, and oral hygiene habits. Several groups were formed based upon the subjects’ answers. (1) The smoking group was comprised of subjects smoking one cigarette or more per day, (2) The habitual use of interdental cleaning tools group was defined as at least one per day, (3) and the tongue scraper group were defined as one use or more per day. The attending dental doctor (K.I.) examined each subject and recorded the number of remaining teeth, presence of dental caries in at least one tooth, score of Community Periodontal Index (CPI) periodontal disease and any diagnosed dental diseases [21, 22]. CPI determination involved division of the oral cavity by subject into six areas and the pocket depth of each tooth was measured and specified for each area via probe using a WHO periodontal probe. CPI were graded based upon the following criteria (0) no sign of disease, (1) gingival bleeding with gentle probing, (2) supra-or subgingival calculus, (3) pathologic pockets 4 or 5 mm deep, and (4) pathologic pockets ≥6 mm. The maximum value for each of the 6 areas was regarded as the CPI of the subject. The severe dental caries group included the diagnostic codes K04, K02.5, and K02.9 of the International Classification of Diseases 10 (ICD-10) [23]. It encompassed caries types that had progressed to the dental pulp and required either dental pulp treatment or tooth extraction. The severe periodontal disease group included periodontitis stage 3 or 4, as defined by “The 2017 World Workshop on the Classification of Periodontal and Peri-Implant Diseases and Conditions”. This new classification system of periodontitis is characterized by a multidimensional staging and grading system [24, 25]. Patients in the severe periodontal disease group required tooth extraction. There mixed group was comprised of subjects having severe dental caries with periodontitis group. The “others” group comprised subjects with tooth fractures group or pericoronitis group.

### Collection of dental pulp, dental plaque, and saliva

Dental pulp samples were collected using sterile root canal instruments and stored in sterile saline in a 10 ml saline kit (Corning Incorporated, Corning, NY, USA). In the case of tooth extraction, teeth were penetrated with a dental air turbine, and the dental pulp was preserved in a 10 ml saline kit. Supragingival plaques were collected from incisors and molar teeth and stored in sterile saline in a 10 ml saline kit. Approximately 5 ml saliva at rest were collected by sterile saline in an Exclusive Collection kit (Salivette; Sarstedt, Nümbrecht, Germany). The samples were immediately transported to our laboratory on ice. They were stored in the laboratory at − 20 °C until further processing.

### Identification of *H. pylori* gene in samples obtained from oral cavity

We used an improved nested PCR protocol that amplified part of the *H. pylori ureA* gene, and detected it in dental pulp, supragingival plaque, and saliva samples [20]. Briefly, 5 ml sample cultures were centrifuged and resuspended in 250 μl of 10 mM Tris/HCl (pH 8.0) and 1 mM EDTA. The cells were collected by centrifugation and lysed in Cell Lysis solution 600 μl at 80 °C for 5 min (Qiagen, Tokyo, Japan). Then, the cell lysate was incubated with 3 μl of RNase (10 mg ml^− 1^) at 37 °C for 30 min (Qiagen, Tokyo, Japan). Following the addition of 200 μl of protein precipitation solution, the lysate was centrifuged, and 600 μl of isopropanol was added to the supernatant (Qiagen, Tokyo, Japan; Wako Pure Chemical Industries, Osaka, Japan). The resulting DNA pellet was washed with 70% ethanol (Wako Pure Chemical Industries, Osaka, Japan), air-dried, and dissolved in 100 μl of Tris-EDTA buffer, and 100 μl of DNA was extracted [10 mM Tris/HCl, 1 mM EDTA (pH 8.0)]. PCR mixture comprised the following: 2 μl of Ex Taw Buffer, 1.6 μl of dNTP Mix, 0.5 μl of Forward primer [MilliQ 80 μl + ureA-aF primer (5′-ATG AAA CTC ACC CCA AAA GA-3′) 20 μl], (40 μg/ml). 0.5 μl of Reverse primer [MilliQ 80 μl + *ureA*-bR primer (5′-TAG ACT TTG ACA GAG AAA CTT TCG G-3′) 20 μl], 0.1 μl of Ex Taq, and 13.3 μl of MilliQ water (Takara Bio Inc., Otsu, Japan). The extracted DNA (2 μl; 20 μg/ml) was added to the mixture (18 μl) and centrifuged. DNA was amplified for 30 cycles by PCR, and the PCR products were separated on a 1.5% (w/v) agarose gel using Tris-acetate-EDTA buffer, stained with ethidium bromide (0.5 μg ml ^− 1^), and visualized under UV illumination (My Cycler™; Bio-Rad, Hercules, California, USA). Nested PCR of the PCR product was performed using the following mixture: 0.5 μl of Forward primer [MilliQ 80 μl + *ureA*-bF primer (5′-AAA CGC AAA GAA AAA GGC ATT AA-3′) 20 μl], 0.5 μl of Reverse primer [MilliQ 80 μl + *ureA*-aR primer (5′-TTC ACT TCA AAG AAA TGG AAG TGT GA-3′) 20 μl]. Likewise, DNA was amplified, and the PCR products were separated as described above.

### Urine collection and detection of *H. pylori* infection

*H. pylori* infection was detected utilizing a urine antibody test (RAPIRUN *H. pylori* Antibody Detection Kit, Otsuka Pharmaceutical Co., Ltd., Tokyo, Japan). Antibody testing was performed immediately after collecting subject urine.

### Statistical analysis

The results for each of the tested factors were compared by gender using the *t*-test or Pearson’s *χ*^2^-test. The odds ratio and *kappa* coefficient were used to analyze the association between *H. pylori* infection and the presence of *H. pylori* in dental pulp. Fisher’s exact test was utilized for analysis of *H. pylori* in dental pulp and severe dental caries. Pearson’s *χ*^2^-test was used to determine the relationship between *H. pylori* in dental pulp and severe periodontal disease. Data analysis with SPSS 19.0 for Windows compared *p*-values and (≤ 0.05) was considered significant (Japan Inc., Tokyo, Japan).

### Research ethics

Our study was approved by the Ethics Committee of Kyoto Prefectural University of Medicine (approval number: ERB-C-473, date of approval: Nov 30th, 2015) and was conducted in accordance with the Declaration of Helsinki ethical principles.

## Results

### Backgrounds of the subjects

Table 1 shows the characteristics, oral examinations, and dental clinical diagnosis for the subjects. Mean age of the subjects 58.6 ± 18.8 years old, with subjects 70 years old and above accounting for 40% (*n* = 76) of the subjects. Subjects who had previously received *H. pylori* eradication therapy accounted for 11% (*n* = 21) of the test group. For the dental characteristics of the subjects, the number of remaining teeth averaged 20.4 ± 6.8, subjects with dental caries were abundant at 57% (*n* = 110), and the percentage of subjects having periodontitis with CPI code ≥3 reached 71% (*n* = 136). Twenty-five subjects in our study were done pulp treatment, and 167 subjects were done tooth extraction. Among the subjects who had severe dental caries, 25 subjects were done pulp treatment and 11 subjects were done tooth extraction. There were no significant gender-related differences in the results of each factor. The percentage of subjects who expressed severe dental caries with periodontitis upon dental clinical diagnosis totaled 30% (*n* = 57), which was markedly higher than other illnesses in our study.

**Table 1:** Characteristics, oral examinations, and dental clinical diagnosis among the subjects

		Males	Females	Total
		*n* = 87	*n* = 105	*n* = 192
Clinical characteristics and oral hygiene habits				
Age average ± SD		59.5 ± 18.9	57.9 ± 20.2	58.6 ± 18.8
Age - grades				
~ 49		26 (30%)	34 (32%)	60 (31%)
50~59		7 (8%)	14 (13%)	21 (11%)
60~69		18 (21%)	17 (16%)	35 (18%)
70~		36 (41%)	40 (38%)	76 (40%)
Body Mass Index (kg/m^2^) ± SD		23.4 ± 4.2	22.6 ± 4.1	23.1 ± 4.0
Smoking		28 (32%)	22 (21%)	50 (26%)
Previous eradication therapy of *H. pylori*		13 (15%)	8 (8%)	21 (11%)
Frequency of teeth brushing (per day) ± SD		2.0 ± 0.5	2.2 ± 0.8	2.1 ± 0.5
Habitual use of interdental cleaning tools +		41 (47%)	47 (45%)	88 (46%)
Habitual use of tongue scraper +		6 (7%)	8 (8%)	14 (7%)
Oral examinations				
Number of remaining teeth ± SD		20.8 ± 7.0	20.4 ± 7.5	20.4 ± 6.8
Dental caries +		53 (61%)	57 (54%)	110 (57%)
Community Periodontal Index ≧ code 3		58 (67%)	78 (74%)	136 (71%)
Dental treatment	Dental clinical diagnosis			
Pulp treatment	Severe dental caries	10 (11%)	15 (14%)	25 (13%)
Tooth extraction	Severe dental caries with periodontitis	20 (23%)	37 (35%)	57 (30%)
	Pericoronitis	32 (36%)	19 (18%)	51 (26%)
	Severe periodontitis	18 (21%)	23 (22%)	41 (21%)
	Severe dental caries	2 (2%)	9 (9%)	11 (6%)
	Tooth fracture	5 (6%)	2 (2%)	7 (4%)

Values are presented as ‘mean ± SD

### The number of subjects with *H. pylori* infection and PCR-positive for *H. pylori* in samples obtained from oral cavity

The proportion of subjects positive for *H. pylori* infection based on the urine antibody test was 13% (25/192) (Table 2). *H. pylori* were either detected in dental pulp (12%, 23/192), or dental plaque (1%, 2/192), but remained undetectable in saliva. The proportion of infection was found to be lower in younger subjects.

**Table 2:** The number of subjects by age grades with *H. pylori* infection and PCR-positive for *H. pylori* in samples obtained from oral cavity

	Presence of *H. pylori*				
	Age – grades				
	20~49	50~59	60~69	70~	Total
	*n* = 60	*n* = 21	*n* = 35	*n* = 76	*n* = 192
Samples					
Urine	0 (0%)	2 (10%)	5 (14%)	18 (24%)	25 (13%)
Dental pulp	0 (0%)	1 (5%)	4 (11%)	18 (24%)	23 (12%)
Dental plaque	0 (0%)	0 (0%)	0 (0%)	2 (3%)	2 (1%)
Saliva	0 (0%)	0 (0%)	0 (0%)	0 (0%)	0 (0%)

### Association between *H. pylori* infection and the presence of *H. pylori* in dental pulp

As shown in Table 3*, H. pylori* infection was determined to be a significant risk factor associated with presence of *H. pylori* in dental pulp (odds ratio = 4.33 × 10^2^, 95% confidence interval: 7.47 × 10–2.50 × 10^3^, *p* <  0.01). The odds ratio showed a very strong correlation regardless of age or score. There was a high correlation between *H. pylori*-infected subjects and the subjects who expressed *H. pylori* in dental pulp (*kappa* = 0.86). Of the 21 subjects who received previous *H. pylori* eradication therapy, three were found to have *H. pylori* in dental pulp, (data not shown in table) and one of these subjects was not infected with *H. pylori*.

**Table 3:** Association between *H. pylori* infection and the presence of *H. pylori* in dental pulp

	*H. pylori* infection	*H. pylori* in dental pulp		OR^a^	95% Cl^b^	*P*-value
		+	–			
Age < 70	+	5/7 (71%)	2/7 (29%)	4.82 × 10^2^	2.06 × 10–1.13 × 10^4^	< 0.05
	–	0/109 (0%)	109/109 (100%)	1.00	ref.	
Age ≧ 70	+	16/18 (89%)	2/18 (11%)	2.24 × 10^2^	2.92 × 10–1.72 × 10^3^	< 0.01
	–	2/58 (3%)	56/58 (97%)	1.00	ref.	
Total	+	21/25 (84%)	4/25 (16%)	4.33 × 10^2^	7.47 × 10–2.50 × 10^3^	< 0.01
	–	2/167 (1%)	165/167 (99%)	1.00	ref.	

^a^Odds ratio (OR)

^b^95% confidence interval (Cl)

### Presence or absence of *H. pylori* in dental pulp on each dental clinical diagnosis

Table 4 was classified by a dental clinical diagnosis among the subjects in our study. Of the 23 subjects expressing *H. pylori* in dental pulp, 66% (15/23) had severe dental caries with periodontitis, 30% (7/23) had severe dental caries only, and 4% (1/23) had severe periodontitis only. Of the subjects having *H. pylori* in their dental pulp, expressed severe dental caries at a significantly higher rate, than any other disease (*p* <  0.01) (Table 5). However, there was no significant difference between severe periodontitis and the other diseases.

**Table 4:** Presence or absence of *H. pylori* in dental pulp on each dental clinical diagnosis

	*H. pylori* in dental pulp	
	+	–
Dental clinical diagnosis	*n* = 23	*n* = 169
Severe dentalcaries with periodontal disease	15 (66%)	42 (25%)
Severe dental caries	7 (30%)	29 (17%)
Severe periodontitis	1 (4%)	40 (24%)
Tooth fracture	0 (0%)	7 (4%)
Pericoronitis	0 (0%)	51 (30%)

**Table 5:** Association between the presence of *H. pylori* in dental pulp and severe dental caries or periodontitis

	*H. pylori* in dental pulp		
	+	–	*P*-value
Dental clinical diagnosis	*n* = 23	*n* = 169	
Severe dental caries	22 (96%)	71 (42%)	< 0.01^a^
Other disease except severe dental caries	1 (4%)	98 (58%)	
Severe periodontitis	16 (70%)	82 (49%)	0.07^b^
Other disease except severe periodontitis	7 (30%)	87 (51%)	

^a^Fisher’s exact test

^b^Pearson’s *χ*^2^-test

## Discussion

In our study, the total proportion of subjects with *H. pylori* infection was 13% (25/192), 24% subjects in their 70-year-old and above, 15% subjects in their 60s, and 8% subjects in their 50s and younger. *H. pylori*-infected subjects plus subjects who had received previous *H. pylori* eradication therapy constituted 24% (46/192) of all subjects. This number was lower than the proportion of *H. pylori* infection in Japanese subjects reported previously [5]. However, the finding that the proportion of *H. pylori* infection is likely to be lower in younger generations is similar to Hirayama’s report.

Our study incorporated an improved nested PCR method targeting the *ureA* gene to identify the presence of the *H. pylori* gene in dental pulp, dental plaque, and saliva of our subjects. In the past, various PCR primers were used to detect *H. pylori* gene in samples obtained from oral cavity. Ogaya et al. developed PCR primers for *H. pylori* along with a new detection method targeting *ureA* [12]. This was accomplished as follows: first, they recovered full DNA sequences for the 48 strains (including Japanese-Korean, South American, Indian, European, and African strains) from GenBank (http://www.ncbi.nlm.nih.gov/genbank/). They then analyzed multiple alignments of the *cagA*, *vacA*, *ureA*, *ureC*, and 16S rRNA genes with CLUSTALW from the DNA Data Bank of Japan (DDBJ) (http://clustalw.ddbj.hig.ac.jp/). As a result, a common sequence of more than 20 consecutive sequences was found only in *ureA*, and this was used to design a PCR primer set for *H. pylori* [12]. Later, this method was improved and used to develop a highly sensitive nested PCR method [20]. Our study incorporated this improved nested PCR method into the study protocol.

The proportion of subjects with *H. pylori* in their dental pulp was 12% (23/192). Our study showed that dental pulp was a reservoir for *H. pylori* in Japanese adults. However, this proportion was lower than that reported by Nomura among Japanese children (1 to 19-year-old, 39%), even though younger generations are likely to have a lower rate of occurrence [20]. This might be attributed to differences in the sampling procedures utilized in the studies. Our study included samples of severe periodontitis and pericoronitis, which were not researched by Nomura et al. Nomura’s report co-cultured human dental pulp fibroblasts and an *H. pylori* strain 26695 (ATCC 700392) (Summit Pharmaceutical Co., Tokyo, Japan), showing that *H. pylori* possessed both adhesion and invasion ability [20]. It therefore can be speculated that *H. pylori* colonized the dental pulp of permanent teeth using this ability.

In contrast to its presence in dental pulp, our study found that the proportion of subjects expressing *H. pylori* in their dental plaque was only 1% (2/192). This was lower than the 25% (14/56), 54% (270/500), and 44% (20/45) reported in previous Japanese, Indian, and Iranian studies, respectively [26–28]. Only supragingival dental plaque was collected in our study, however the Japanese report by Umeda et al. was not limited in this way [26]. In the Indian report, the proportion of *H. pylori* infection was 69% (in our study it was 13%). In that study, sewage facilities and waste treatment facilities were not available to 18 and 17% of the areas in which subjects lived, respectively [27]. The Iranian report included poor hygiene areas, and all subjects were found to be infected with *H. pylori* [28].

*H. pylori* was not detected in saliva samples in our study. Previously, *H. pylori* had been found in saliva from 6% (21/326) of subjects in another Japanese report, 30% (9/30) in a Brazilian report, and 8% (25/300) in an Iranian report [10, 29, 30]. Furthermore, all of the subjects in the Brazilian and Iranian reports were infected with *H. pylori* [10, 30]. The low proportion of *H. pylori* infection found in our study might have a large impact on the difference in the subjects who were found to have *H. pylori* in dental plaque and saliva compared to previous studies. Previously, a Japanese report by Ogaya et al. found no *H. pylori* in saliva from Japanese children in an area geographically similar to that of our study [12]. Therefore, *H. pylori* infection can be expected to be influenced by geographical area, hygiene, and oral states. In Japanese hygienic environments and with today’s oral hygiene practices, microbial flora would be present in supragingival plaque and saliva only temporarily, and *H. pylori* may have difficulty accumulating [31].

*H. pylori* infection was a significant risk factor for the presence of *H. pylori* in dental pulp, regardless of age or score. Almost all *H. pylori* infection occurred within 5 years after birth [32]. Since immune mechanism is established in the adult stomach, invasion of *H. pylori* in adults only caused acute gastritis, and that the stomach is not easily colonized [33]. In the report by Hirsch et al., viable *H. pylori* was detected in the root canal of deciduous teeth [27]. Our study found that *H. pylori* travels back through the esophagus to the oral cavity after the permanent dentition is replaced, and then accumulates in the dental pulp of permanent teeth. Viable *H. pylori* in dental pulp might be involved in reinfection of the stomach. On the other hand, among the *H. pylori*-infected subjects, the proportion who did not have *H. pylori* in their dental pulp was 16% (4/25). These four subjects had severe dental caries in other teeth. Therefore, they might have had *H. pylori* in the dental pulp of the untested teeth. Only two subjects among the *H. pylori* non-infected subjects had *H. pylori* in their dental pulp. In these two, *H. pylori* might have colonized the oral cavity directly from outside. One of these two subjects had previously received *H. pylori* eradication therapy. Because antibiotics do not easily penetrate dental pulp [34], *H. pylori* in dental pulp might remain viable after eradication.

Severe dental caries were significantly more common than other diseases among subjects who had *H. pylori* in their dental pulp. However, there was no significant difference between subjects with severe periodontitis and other diseases. These results suggest that *H. pylori* is transmitted from carious cavities to the root canal, where colonization occurred in the dental pulp. Carious cavities are difficult to clean using a brush, and the microbial flora does not usually change. Therefore, they constitute a suitable reservoir for *H. pylori* colonization. Prevention of dental caries and early treatment of dental caries will decrease the reservoir of *H. pylori* in the oral cavity, and likely prevent *H. pylori* reinfection of the stomach following eradication.

Our study had several limitations. Firstly, subgingival plaque was not collected. It is not clear whether *H. pylori* accumulated in the periodontal pocket or whether it infected the root canal and dental pulp from the periodontal pocket. In addition, the timing of dental caries development was not confirmed because this was a cross-sectional, and not a longitudinal, study. Duration and severity of dental caries might influence the presence of *H. pylori* in dental pulp. In the future, the collection of subgingival plaque and longitudinal study would be necessary. Finally, *H. pylori* strains in the stomach and dental pulp were not confirmed to be identical. In future work, the Authors will work to further elucidate these strains.

## Conclusions

Our study found that many Japanese adults who were infected with *H. pylori* tested positive for the presence of the *H. pylori ureA* gene in samples obtained from oral cavity. Furthermore, dental pulp, rather than dental plaque or saliva, was shown to be the main reservoir for *H. pylori* in the oral cavity. Further, *H. pylori* infection is likely transmitted from carious cavities in the root canal to the dental pulp.

## Data Availability

The datasets used and analyzed during the current study are available from the corresponding author on reasonable request.

## Abbreviations

*CFU*: *Colony forming units*
*H. pylori*: *Helicobacter pylori*
